# Serum Biomarkers to Dynamically Predict the Risk of Cardiovascular Events in Patients under Oncologic Therapy. A Multicenter Observational Study

**DOI:** 10.31083/j.rcm2507256

**Published:** 2024-07-09

**Authors:** Nicoletta Provinciali, Marco Piccininno, Giacomo Siri, Alessandra Gennari, Giancarlo Antonucci, Damiano Ricci, Emmanuela Devoto, Roberta Miceli, Pietro Cortesi, Chiara Pazzi, Oriana Nanni, Francesca Mannozzi, Ilaria Pastina, Luciana Messuti, Carmelo Bengala, Giovanni Luca Frassineti, Carlo Cattrini, Marianna Fava, Tania Buttiron Webber, Irene Maria Briata, Davide Corradengo, Andrea DeCensi, Matteo Puntoni

**Affiliations:** ^1^Division of Medical Oncology, Ente Ospedaliero Ospedali Galliera, 16128 Genoa, Italy; ^2^Department of Experimental Medicine, University of Genoa, 16126 Genoa, Italy; ^3^Cardiology Unit, Ente Ospedaliero Ospedali Galliera, 16128 Genoa, Italy; ^4^Clinical Trial Unit, Office of the Scientific Director, Ente Ospedaliero Ospedali Galliera, 16128 Genoa, Italy; ^5^Division of Oncology, Maggiore della Carità University Hospital, 28100 Novara, Italy; ^6^Department of Traslational Medicine, University of Piemonte Orientale, 28100 Novara, Italy; ^7^Internal Medicine Unit, Ente Ospedaliero Ospedali Galliera, 16128 Genoa, Italy; ^8^Oncology Unit, Istituto Romagnolo Per Lo Studio Dei Tumori “Dino Amadori” (IRST) IRCCS, 47014 Meldola, Italy; ^9^Biostatistics and Clinical Trial Unit, Istituto Scientifico Romagnolo per lo Studio e la Cura dei Tumori (IRST), IRCCS, 47014 Meldola, Italy; ^10^Oncology Unit, Ospedale Misericordia, 52100 Grosseto, Italy; ^11^Wolfson Institute of Population Health, Barts and the London School of Medicine and Dentistry, Queen Mary University of London, E1 2AD London, UK; ^12^Clinical and Epidemiological Research Unit, University Hospital of Parma, 43126 Parma, Italy

**Keywords:** cardio-oncology, anticancer therapies, cardiovascular toxicity, STEPP analysis, oxidative stress biomarkers

## Abstract

**Background:**

Serum biomarkers have been investigated as predictive risk factors for cancer-related cardiovascular (CV) risk, but their analysis is limited to their baseline level rather than their overtime change. Besides historically validated causal factors, inflammatory and oxidative stress (OS) related markers seem to be correlated to CV events but this association needs to be further explored. We conducted an observational study to determine the predictive role of the longitudinal changes of commonly used and OS-related biomarkers during the cancer treatment period.

**Methods:**

Patients undergoing anticancer therapies, either aged 75+ years old or younger with an increased CV risk according to European Society of Cardiology guidelines, were enrolled. We assessed the predictive value of biomarkers for the onset of CV events at baseline and during therapy using Cox model, Subpopulation Treatment-Effect Pattern Plot (STEPP) method and repeated measures analysis of longitudinal data.

**Results:**

From April 2018 to August 2021, 182 subjects were enrolled, of whom 168 were evaluable. Twenty-eight CV events were recorded after a median follow up of 9.2 months (Interquartile range, IQR: 5.1–14.7). Fibrinogen and troponin levels were independent risk factors for CV events. Specifically, patients with higher than the median levels of fibrinogen and troponin at baseline had higher risk compared with patients with values below the medians, hazard ratio (HR) = 3.95, 95% CI, 1.25–12.45 and HR = 2.48, 0.67–9.25, respectively. STEPP analysis applied to Cox model showed that cumulative event-free survival at 18 and 24 months worsened almost linearly as median values of fibrinogen increased. Repeated measure analysis showed an increase over time of D-Dimer (*p*-interaction event*time = 0.08), systolic (*p =* 0.07) and diastolic (*p =* 0.05) blood pressure and a decrease of left ventricular ejection fraction (*p =* 0.15) for subjects who experienced a CV event.

**Conclusions:**

Higher levels of fibrinogen and troponin at baseline and an increase over time of D-Dimer and blood pressure are associated to a higher risk of CV events in patients undergoing anticancer therapies. The role of OS in fibrinogen increase and the longitudinal monitoring of D-dimer and blood pressure levels should be further assessed.

## 1. Introduction 

The American College of Cardiology and American Heart Association guidelines [1] recommend to consider patients undergoing antineoplastic treatments at increased risk of developing a cardiac dysfunction.

The increased incidence of cardiovascular (CV) events is due on the one hand to the toxicity of the treatments themselves and on the other one to the longer survival of the patients [2]. The CV damages can be very different depending on the time of onset and duration, so that we can distinguish acute, sub-acute or chronic toxicities; moreover, adverse events vary substantially according to the antineoplastic agent that caused them. Anthracyclines may be responsible for ventricular dysfunction [3], fluoropyrimidines can cause acute myocardial ischemia [4], anti-human epidermal growth factor receptor 2 (HER2) agents are involved in left ventricular dysfunction [5], tyrosine kinase inhibitors and anti-vascular endothelial growth factors are related to the onset of arterial hypertension [6, 7]. More recently, the most frequently reported CV event related to immune checkpoint inhibitors is the onset of myocarditis [8].

In addition, other risk factors for CV events are the duration and dose of drugs exposure and patients-related factors. These latter, such as age, gender, smoking, cholesterol levels and blood pressure [9] are essentials, together with the presence of already known CV or metabolic diseases, to assign patients to different CV risk classes, in accordance to the European Society of Cardiology guidelines [10]. The risk to develop an anti-cancer treatment-related toxicity is higher in elderly patients and in those with an increased CV risk even before starting therapy [11, 12].

Anti-cancer drugs related CV adverse events (e.g., arrhythmia, hypertension, thromboembolic or vascular disease, stroke, *etc*.) [13] are one of the most common causes of treatment discontinuation. Therefore, it is necessary to treat these patients in the best and most timely way.

To date, the traditional approach to monitor heart function is based on the left ventricular ejection function (LVEF) assessment by echocardiography [1, 14]. However, a clinically significant change in heart function is detectable only after the onset of a CV event and has limited predictive value.

Recently, a correlation was found between CV events and oxidative stress (OS)/inflammation-related markers [15] (e.g., C-reactive protein, Interleukin-6, fibrinogen). Myeloperoxidase (MPO) is the main source of OS. When vascular damage occurs, neutrophils and macrophages in the vasculature overproduce MPO, which contributes to the formation of atheromatous plaques in the vessel walls with the production of reactive oxidant species (ROS), which induce plaques dissection [16].

So, MPO can be used as an early diagnostic and prognostic marker for the onset of some CV diseases. In fact, high levels of MPO identified high-risk patients according to the Global Registry of Coronary Artery Events (GRACE) scores [17]. In these patients, a correlation between MPO levels and inflammatory markers, such as fibrinogen, has been proved. Moreover, endothelial (e.g., Albumine-to-creatine ratio), renal function (creatinine) and coagulation biomarkers (e.g., D-dimer) may predict CV risk in addition to high-sensitive troponins (hs-Tn) [18] and natriuretic peptide (N-terminal-pro hormone brain natriuretic peptide (BNP)) [19]. However, only troponins and BNP are currently recognized as potential predictors of CV toxicities [14]. The main limitations of the current algorithms are the use of the only baseline information, collected before the start of therapy. A predictive approach using the historical evolution of biomarkers collected longitudinally during treatment could be useful to calculate a dynamic estimate of CV risk and to identify and promptly treat patients at high risk of complications. This could end in a better prognosis and a higher quality of life, in addition to allow the normal continuation of antineoplastic therapy. The aim of this study is to explore the predictive role of a set of commonly used and OS-related biomarkers, considering the change of their value from baseline over time during antineoplastic treatment. For the identification of patients with an increased risk of CV events based on the change of their circulating biomarkers during treatment, we adopted repeated measure analysis through mixed effect modelling and the Subpopulation Treatment-Effect Pattern Plot (STEPP) technique [20]. The longitudinal monitoring of each biomarker represents an innovation compared to the traditional approach that uses only baseline data.

## 2. Materials and Methods

### 2.1 Study Design and Target Population

CARIOCA (CArdiovascular RIsk of OncologiC therApy) study is a multicenter observational study with mixed study population formed of, both retrospective and prospective patients. This type of design was possible because all the planned assessments are part of the current clinical practice in all of the three specialized centers involved in the study, where a specific CV monitoring for patients receiving cancer therapy is already ongoing. Target population consisted of adult cancer patients treated with antineoplastic drugs that could predispose to an increased risk of CV events, either because aged over 75 years or ≤75 with moderate/high CV risk according to European Society of Cardiology (ESC) guidelines [14]. In particular, risk was defined “moderate” in the presence of at least two of the following conditions: age (men >55, women >65 years); cigarette smoke; dyslipidemia (total cholesterol >190 mg/dL or LDL cholesterol >130 mg/dL or triglycerides >150 mg/dL or high density lipoprotein (HDL) cholesterol <40 mg/dL for men and <50 mg/dL for women); fasting hyperglycemia (>100 and <126 mg/dL); family history of early CV event (first-degree relatives, men <55 years, women <65 years); obesity (waist circumference >102 cm for men, >88 cm for women). Risk was defined “high” in the presence of at least one of the following conditions: severe hypertension (di-astolic pressure ≥110; systolic pressure ≥180 mmHg); diabetes mellitus (or baseline blood glucose ≥126 mg/dL); previous cardio/cerebro or peripheral vascular event; evidence of organ damage.

Eligible drugs for the enrollment in the study were: anthracyclines, docetaxel, paclitaxel, cisplatin, 5-fluorouracil (5FU), capecitabine, trastuzumab, bevacizumab, pertuzumab, sorafenib, sunitinib, pazopanib, lapatinib, axitinib, regorafenib, everolimus, tamoxifen and abiraterone. These drugs are known to be associated with CV toxicity, according to 2020 European Society of Medical Oncology (ESMO) consensus recommendations [21].

To be enrolled in the study, patients should have at baseline a normal cardiac function (LVEF ≥50%). Alterations of BNP values were accepted if minimal and considerable physiologically related to the age of subjects.

Exclusion criteria were previous treatment with the mentioned above anti-cancer drugs and the presence of ongoing CV diseases, such as heart failure (i.e., LVEF <50%) or presence of symptoms, uncontrolled arterial hypertension, unstable angina pectoris, cardiac arrhythmias associated with symptoms or CV instability or psychiatric diseases.

Inclusion and exclusion criteria are reported in **Supplementary Table 1**.

### 2.2 Primary Endpoint

The primary endpoint measure was composite and represented by the onset of at least one CV event. The CV toxicities considered for the study included ischemic events, heart failure, blood pressure rises, kidney damage (**Supplementary Table 2**).

### 2.3 Secondary Endpoints

Secondary endpoints measures were the serum biomarkers levels which were measured at baseline, before the beginning of the treatment, during the therapy and, finally, during the follow-up period after the last cycle of therapy as defined in the monitoring plan.

Standard biochemical and metabolic profile biomarkers, the assessment of LVEF (two-dimensional echocardiography - Simpson-Biplane method), 24 hour blood pressure monitoring (BPM Holter) and the assessment of the CV risk were evaluated, according to ESC guidelines [14], at each time point. In addition, the main serum biomarkers included in our study were the following: high-sensitive (hs) troponin T or I [22, 23, 24, 25]; N-terminal proB-type natriuretic peptide (NT-proBNP) [26, 27]; Fibrinogen [28, 29, 30]; D-dimer [31, 32]; C-reactive protein [23, 33]; Albumin to creatinine ratio (ACR) [34, 35]; type, administration dose and duration of anticancer therapy were be also recorded. The correlation between changes in these biomarkers overtime and the onset of CV events was analyzed.

### 2.4 Sample Size

Given that the incidence of CV toxicity (as previous defined) in cancer patients undergoing antineoplastic treatment is about 5–10% (>15% in patients treated with anthracyclines), we initially calculated that a total of 200 patients to be enrolled in 2 years, plus 1 year of follow-up, would have been sufficient to observe a total of 40 CV events, which would had provided an adequate power (80%) to assess risk factors with a relative risk of event higher than 2.0.

### 2.5 Statistical Methods

Data were explored by using the common descriptive statistics such as mean and standard deviation, median and interquartile range (IQR) for continuous variables or absolute and relative frequency (%) for categorical variables. Based on the distance from normality of biomarkers distribution, we adopted quantiles (quartiles or medians) as cut-off values in the analyses. The onset of a CV event was evaluated primarily by using a classic “time to event” approach (i.e., Kaplan-Meier curves and Cox Proportional Hazard model) and a more complex Cox model with time-varying information acquired during the treatment (change and suspension of therapy), to study the possible variation in the effect on CV toxicity over time. Event-free survival (EFS) was defined as the time from the start of antineoplastic treatment and the date of a CV event or the date of last follow-up visit. STEPP methodology [20] was used to explore and display graphically how the risk of CV events changes as a function of the continuous scale of biomarkers, along the continuous scale of the biomarkers levels [36], using overlapping subject subgroups. The impact of longitudinal biomarkers changes on the CV risk prediction was addressed through a mixed effects model, which allows accounting for the correlation between repeated measurements of biomarkers collected on the same patients. We adopted as fixed effects age, sex, enrollment center, CV risk (high vs. medium/low) and CV event. The random effects were the intercept variance and the residual variance, which correspond to the between-subjects and within-subjects variances, respectively. To evaluate the difference of linear trend over time of biomarkers in subjects who had a CV event compared to those who did not, we assessed the interaction term between the CV event (yes/no) and time elapsed since the visit baseline to biomarker measurement: we considered as worthy of being evaluated *p*-values for interaction <0.2. Given the exploratory nature of the study, no correction technique for multiplicity was adopted and a 2-tailed alpha error of 5% was considered as a cut-off value to declare the statistical significance of the tests used. We included all participants for whom the variables of interest were available in the final analysis, without imputing missing data. Results are shown with 95% confidence intervals (95% CI) and statistical significance was considered for two-sided *p* values < 5%. All analyses were performed using STATA, version 17.0 (StataCorp, College Station, TX, USA).

## 3. Results

**Fig. 1.**
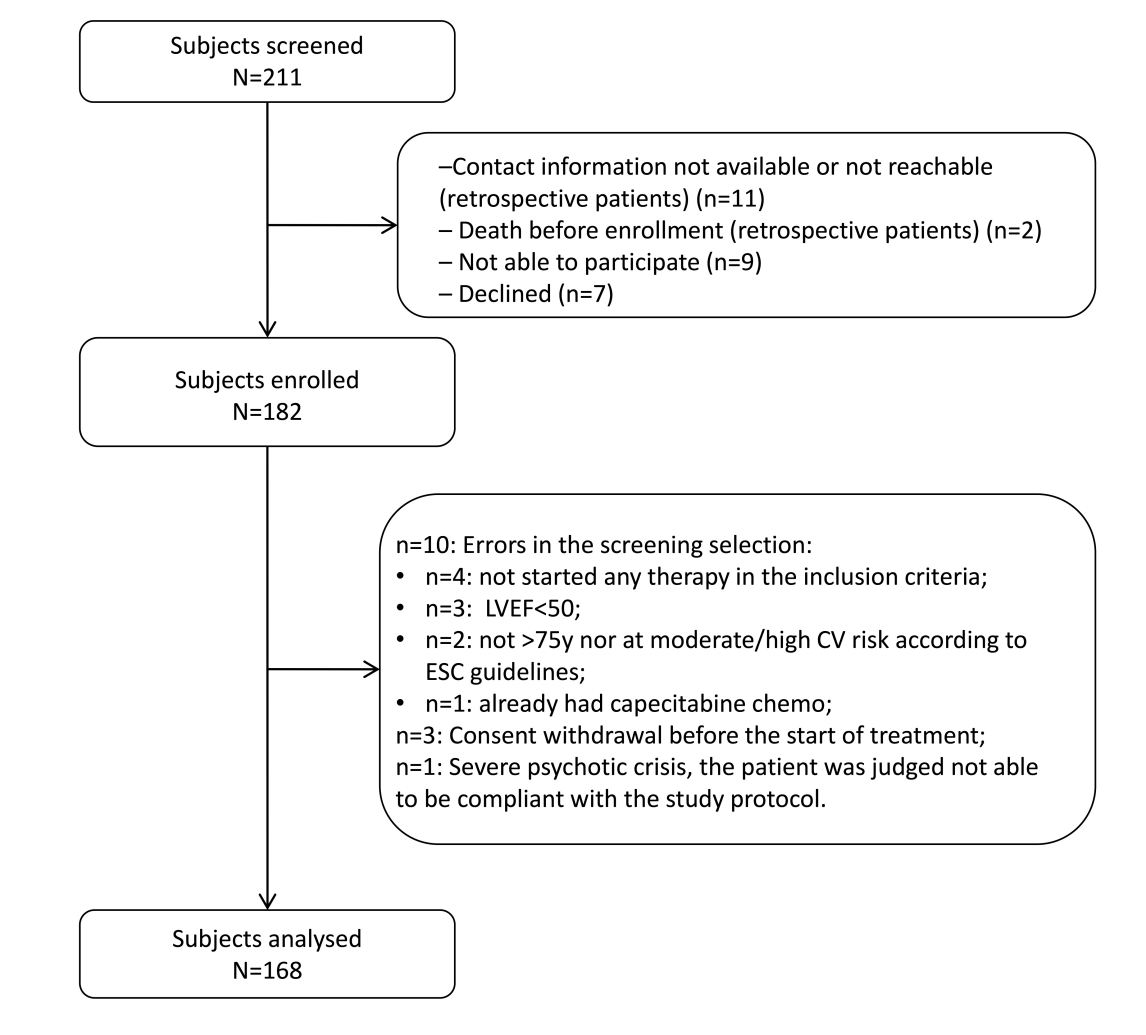
**Flow-chart diagram of the study. **Abbreviations: LVEF, left ventricular ejection fraction; y, years; CV, cardiovascular; ESC, European Society of Cardiology.

From April 2018 to August 2021, 182 subjects were enrolled, of whom 168 were evaluable (Fig. 1).

The enrollment phase was slower than expected for the COVID-19 pandemia so we had to stop it before reaching the expected number of subjects. Detailed enrolled patients’ characteristics are showed in Table 1.

**Table 1.** **Subjects’ characteristics at baseline (n = 168)**.

Sex, n (%)		
	Male	75 (44.6)
	Female	93 (55.4)
Age, mean (SD)		70 (9.9)
Tumor type, n (%)		
	Colorectal	47 (27.8)
	Breast	36 (21.4)
	Lung	7 (4.2)
	Kidney	19 (11.3)
	Ovary	12 (7.1)
	HCC	4 (2.4)
	Other sites	43 (25.6)
ECOG Performance status, n (%)		
	0	103 (61.3)
	1	13 (7.7)
	Unknown	52 (31.0)
Stage of cancer, n (%)		
	Metastatic	94 (56.0)
	*In situ*	45 (26.8)
	Locally advanced	29 (17.3)
BMI, ${\mathrm{kg/m}}^{2}$, median (IQR)		25.0 (22.7–27.6)
Cardiovascular Risk*		
	No Risk	6 (3.6)
	Low Risk	35 (20.8)
	Medium Risk	76 (45.2)
	High Risk	51 (30.4)
Previous antineoplastic treatment		
	No	140 (83.3)
	Yes	28 (16.7)
Antineoplastic agent		
	Bevacizumab	32 (19.1)
	Taxanes	30 (17.9)
	TKIs	28 (16.8)
	Trastuzumab	24 (14.3)
	5–Fluorouracil/Capecitabine	17 (10.2)
	Cisplatin	16 (9.5)
	Anthracyclines	10 (6)
	Others	7 (4.2)
	Abiraterone Acetate	4 (2.4)
Arterial pressure, mmHg		
	Systolic	130 (120–140)
	Diastolic	80 (70–80)
LVEF, %		63 (60–68)

Abbreviations and notes: SD, standard deviation; HCC, hepatocellular carcinoma; ECOG, Eastern Cooperative Oncology Group; IQR, interquartile range; TKIs, tyrosine kinase inhibitors; *see text for the definition of cardiovascular risk categories; LVEF, left ventricular ejection fraction; BMI, body mass index.

The mean age was 70 years, and 55% of patients were females. The most frequent tumors were colorectal or breast cancers (28% and 21% respectively), followed by kidney (11%), ovary (7%), lung (4%) and HCC (2%). After a median follow-up of 9.2 months (interquartile range, IQR: 5.1–14.7) a total of 28 CV events occurred (**Supplementary Table 3**).

**Fig. 2.**
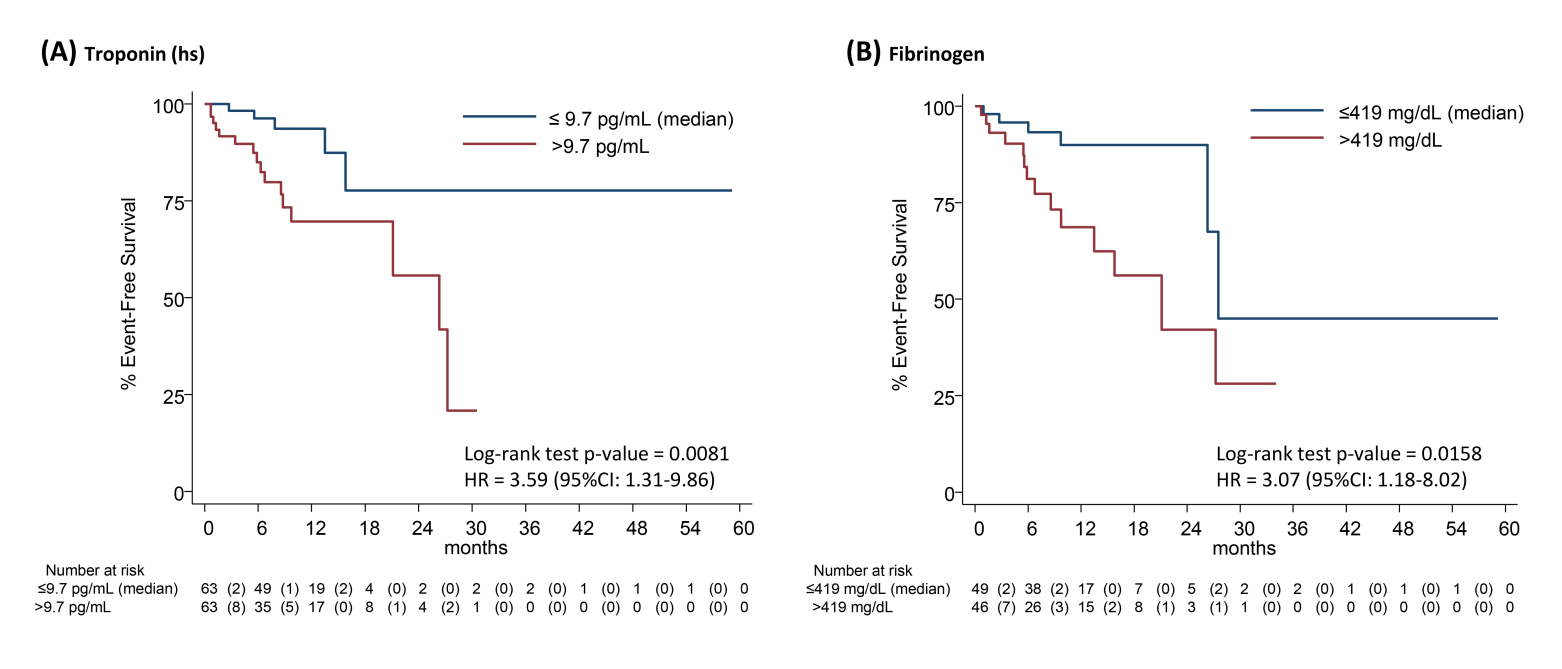
**Kaplan-Meier estimates of cumulative event-free survival rate, by median levels of Troponin (hs) (panel A), and of Fibrinogen (panel B).** Numbers in parentheses represent the number of events within each time interval. The estimate of the hazard ratio (HR) was based on a univariate Cox proportional hazards regression model. hs, high-sensitivity.

Among all the biomarkers analyzed (**Supplementary Table 4**), time-to-event Kaplan-Meier analyses showed significant log-rank *p*-values only considering the median levels of troponin-T (*p* = 0.008) and fibrinogen (*p* = 0.016) at baseline. Kaplan-Meier curves and univariate hazard ratios (95% CI) are shown in Fig. 2.

Multivariable Cox model was used to evaluate the predictive value of risk biomarkers at baseline. A value of fibrinogen above the median of 419 mg/dL, emerged as an independent factor significantly associated with the composite CV event (Table 2). Main subjects’ characteristics, by fibrinogen median value at baseline, are reported in **Supplementary Table 5**.

**Table 2.** **Multivariable Cox model**.

		Hazard ratio	95% CI	*p*-value
Troponin-T, *pg/mL*				
	≤9.7 (median)	1.00		
	>9.7	2.63	0.65–10.56	0.174
Fibrinogen, *mg/dL*				
	≤419 (median)	1.00		
	>419	3.53	1.06–11.77	0.040

Notes: Hazard ratios are adjusted for age, sex and enrollment centre. Harrell’s C concordance statistic of the model = 0.8083. Abbreviations: 95% CI, 95% confidence interval.

Besides, a value of Troponin-T higher than the median of 9.7 pg/mL was strongly associated, even if not in a statistically significant manner: the Cox model estimated an approximately 3.5-fold increased risk for fibrinogen (hazard ratio (HR) = 3.53, 1.06–11.77), and 2.5-fold for troponin (HR = 2.63, 0.65–10.56), compared to the risk of subjects with values below the median at the baseline visit.

**Fig. 3.**
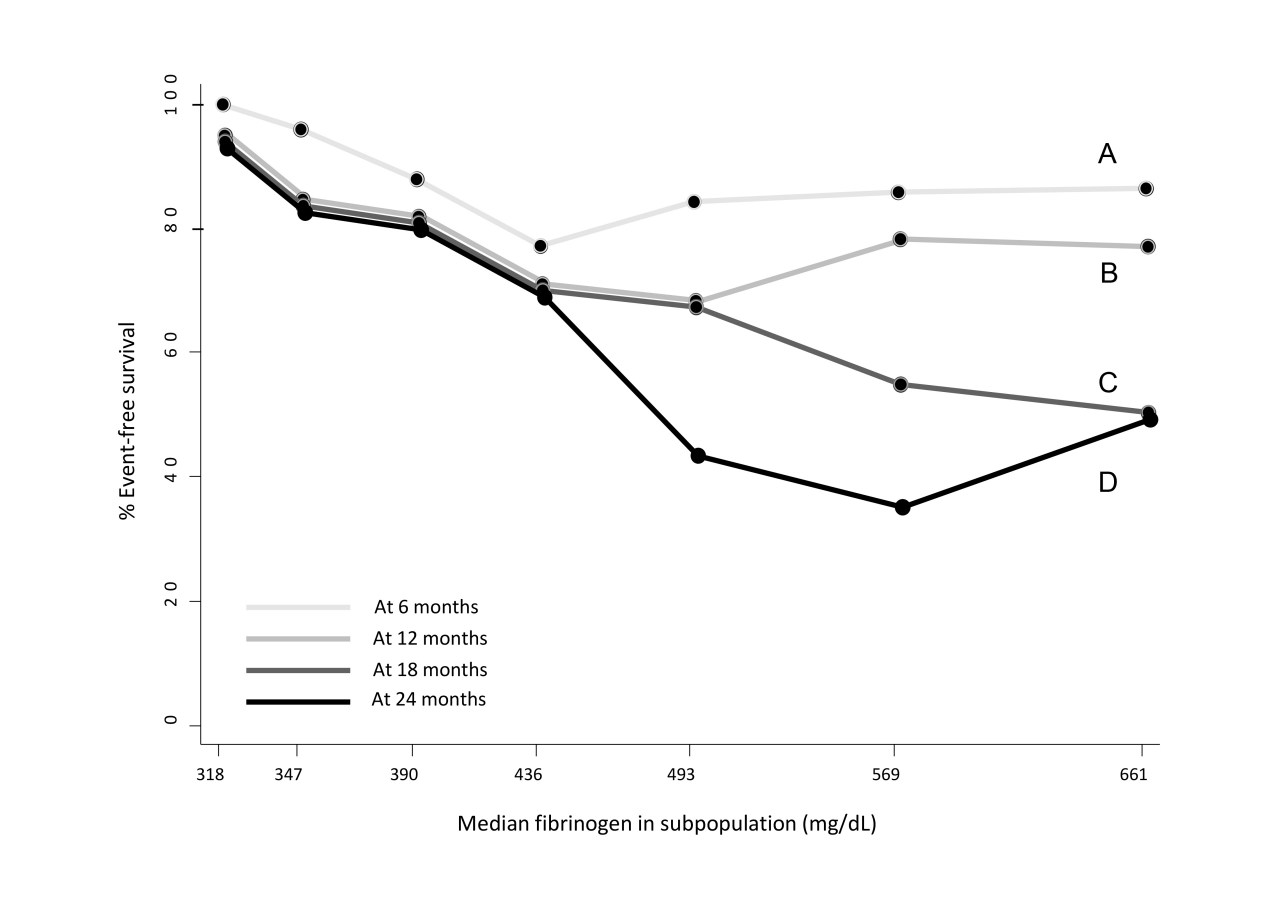
**Subpopulation Treatment Effect Pattern Plot (STEPP) of the 6-months (A), 9 months (B), 18-months (C), 24-months (D) cumulative CV event free-survival rate. **The plot was drawn adopting the sliding window pattern, considering 7 consecutive subpopulations of n = 25 subjects, with n = 15 subjects in common. CV, cardiovascular.

The STEPP graphical technique applied to the time-to-event data clearly showed a relationship between subpopulations with increasing fibrinogen levels and decreased event free survival over time (Fig. 3).

There was a trend for EFS up to 12 months to almost linearly decrease for values slightly below the median of fibrinogen level at baseline (<436 mg/dL) and then plateau at EFS = ~80% for higher values. Conversely, for longer follow-up there was a cumulative effect with a dramatic drop of EFS up to ~40–60% for fibrinogen levels higher than the median at baseline.

During the study period a total of 81 (48%) patients discontinued treatment. Of these, 37 (46%) changed, 25 (31%) discontinued, and 19 (23%) both changed and discontinued therapy. 14 of 28 patients who experienced the CV event (50%) discontinued treatment after the CV event. A consequential link between the CV event and the variation of the treatment was observed in 9 of these patients: 7 changed and 2 definitely discontinued the ongoing treatment which was considered cause of the CV event. The other 5 patients changed or discontinued therapy due to disease progression.

To estimate the prognostic value, in terms of CV event, of the change or suspension of therapy of subjects enrolled in study, we treated them as time-varying covariates using the “stsplit” command of STATA. The dataset was restructured in order to separately take into account the observation times of each subject who had a change in treatment and/or a suspension of therapy. Therefore, for each subject who had a change in treatment or suspension of therapy before the CV event a new record was generated and it was thus possible to run the Cox model considering the “time dependence” of these covariates. Actually, their parameters in the model resulted to be far from the statistical significance and not able to modify the parameters of other factors included in the model, i.e., fibrinogen and troponin. Thus, to reduce model complexity and keep stable risk estimates, we decided to exclude them from the final model. Levels of biomarkers at baseline are described in **Supplementary Table 4**. We longitudinally collected biomarkers levels for a total of 1314 measurements on the 168 patients enrolled (median number of measurements per subject: 5, IQR: 2–8). For some of the biomarkers, the mixed model for repeated measures on longitudinal data showed a different linear trend over time between subjects who experienced a CV event vs. those who did not. Specifically, we found an increase over time of D-dimer (*p* for interaction time x event = 0.084), systolic (*p*-interaction = 0.071) and diastolic (*p* = 0.050) blood pressure values, while a decrease of LVEF (*p* = 0.154) values for subjects who developed a CV event (Table 3).

**Table 3.** **Mixed model for longitudinal biomarkers data**.

	Coeff.*	95% CI	*p*-value
D-Dimer	108.97	–14.48, 232.42	0.084
Systolic blood pressure	0.43	–0.04, 0.90	0.071
Diastolic blood pressure	0.27	0.00, 0.54	0.050
LVEF	–0.15	–0.36, 0.06	0.154

Notes: *coefficient of the interaction term “time x event CV”. Abbreviations: 95% CI, 95% confidence interval; LVEF, left ventricular ejection fraction; CV, cardiovascular.

Predicted linear trends are graphically depicted in **Supplementary Fig. 1**.

## 4. Discussion

This study assessed the onset of CV events in patients undergoing cancer therapies. Changes in biomarkers levels over time have been evaluated as possible predictive risk factors. A total of 28 out of 182 enrolled patients (about 15%) developed an event, confirming that cardiotoxicity is a rather common occurrence and that any possible prevention method to decrease risk needs to be investigated.

Moreover, among these 28 patients, we observed 9 (32%) variations (7 changes and 2 discontinuations) of treatment directly due to the CV event. This evidence strengthens the need of finding a useful tool for early establishing the risk of the onset of a CV event, to prevent this event from influencing the patient’s therapeutic history.

The most significant finding obtained from our study is the identification of two independent variables related to an increased risk of developing a CV event for patients undergoing antineoplastic therapy: high values (above the median in our cohort) of troponin and fibrinogen. Troponin and BNP are the only two biomarkers whose serial dosage is universally recognized for early identification of the onset of cardiac damage [22, 23, 24, 25, 26, 27]. In particular, an increase in troponins is a sign of cardiomyocyte necrosis and above all high values of troponin I are able to reveal cardiac toxicity before it is clinically significant and the damage becomes irreversible [37]. In cancer patients setting, this correlation is observed mostly for those undergoing anthracyclines and trastuzumab [18]. Since it has been shown that in advanced stage tumors high values of troponins and BNP can be recorded even before starting chemotherapy [38], the observation of its increase over time, during and after oncological therapy, seems to be more effective rather than a punctual assessment of troponin at baseline [23, 39]. Our study is in line with this approach: although not formally statistically significant, approximately 3-fold increased risk of CV event for patients in whom an increase in troponin has been observed over time.

Interestingly, we found an even stronger association between high fibrinogen levels and CV risk, with a 3.5-fold increased onset of CV events. This association can be explained by the fact that fibrinogen is considered a marker of inflammation, which is both a cause and a consequence of OS [40]. Oxidative Stress is in turn not only a direct cause of CV diseases [41], but also of other pathological conditions predisposing to CV, such as diabetes [42], obesity [43] and metabolic syndrome [44]. With reference to the latter, outside the oncology setting, increased plasma fibrinogen levels have been demonstrated in subjects with metabolic syndrome [45]. Moreover, therapeutic strategies for cancer, in particular chemotherapy and hormone treatments, can induce alterations in the metabolic pattern of patients, which may induce an increased risk of occurrence of CV events [46, 47].

Oxidative Stress is a pathological condition consequent to an accumulation of ROS due to an increase in their production, as precisely occurs in inflammation, and a reduction in their elimination. Furthermore, it has been demonstrated that during cancer treatment there is a reduction of total radical antioxidant parameters [48]. Our finding of an increasing cumulative risk of CV event at higher levels of fibrinogen and longer follow-up strengthen the hypothesis that fibrinogen is a marker of a major OS due to the chronic inflammatory condition present in cancer patients, together with a reduced antioxidant defense. The consequent accumulation of ROS could explain an easier predisposition to develop CV events.

We observed a reduction in LVEF in patients who experienced a CV event, confirming that monitoring systolic function via repeated echocardiograms over time is a useful strategy. Historically, this was the most used test to assess cardiotoxicity of anti-cancer drugs but has limited predictive value [1, 14], whereas more recently developed myocardial mechanic parameters, such as global longitudinal strain (GLS), have emerged as an effective method for early detection of systolic dysfunction [49]. A reduction of GLS >15% during the treatment is the cut-off value for suspecting cardiac dysfunction despite the absence of symptoms [50].

In our patients, we observed a correlation between an increase in systolic blood pressure (SBP) and diastolic blood pressure (DBP) and the onset of a CV event. Hypertension is a well-known side effects related to oncological therapies, in particular to vascular endothelial growth factor inhibitors [51], but its onset may also be related to the use of ancillary drugs such as steroids, which usually are associated with the antineoplastic treatment [52]. Recently, OS has recently been identified as one of the pathophysiological mechanisms underlying hypertension, which in turn induces OS on the arterial wall, exerting an atherogenic mechanism [53].

We observed an increase in the value of the D-dimer in patients who underwent a CV event. This correlation is known for healthy patients [31, 32], but a recent study underlined this association [54] also in cancer patients setting the hypothesis already validated by a larger trial which demonstrated how the increase in D-dimer values is related to heart failure with preserved ejection fraction [19].

Alongside “classical” biomarkers, others which are related to inflammation and metabolism are emerging as factors able to predict and early detect the onset of CV toxicity in patients undergoing cancer treatments, but further in-depth studies are needed [55, 56].

This is an observational study based on real life clinical practice, with some consequent limits. Enrolled subjects have very variable characteristics due to the type of tumor, the stage of the disease, the type of drugs administered and the different lines of therapy they have taken. Certainly, the heterogeneity of the population and of the levels of the biomarkers measured is one of the major limitations of this study which could be an issue of generalizability. This is partly due to the fact that follow-up parameters, collected according to clinical practice, were referred to both prospective and retrospective data.

Anyway, we are convinced that our findings put the basis for a more in-depth study of the correlation between a progressive increase in serum fibrinogen and the onset of CV events, suggesting to routinely include its measurement before, during and after chemotherapy, also considering that this parameter is easily detectable and inexpensive.

The predictive algorithms currently available for estimating CV risk during oncologic therapy use only information obtained before the beginning of the treatment. This results in a poor discriminating and predictive power and the difficulty to update the risk prediction during therapy (“dynamic” prediction). The approach we propose, on the contrary, should lead to new and dynamic tool that incorporate all the information acquired during the treatment, providing the clinicians with continuous update of the risk estimates for the CV risk.

## 5. Conclusions

The definition of a model able to estimate a CV risk based on follow-up data could influence physicians’ decision making and impact on the quality of life of patients. Our study highlights the role of fibrinogen and troponin as predictive factors of CV event for patients undergoing antineoplastic treatments. Moreover, it might be useful to closely monitor the D-dimer values together with the blood pressure and the LVEF during the therapy as, over time, they move in a significantly different way for patients who will develop a CV event and those who will not.

The use of longitudinal information able to personalize the risk assessment and prediction during antineoplastic therapy should have immediate impact on the decision-making of clinicians and on prognosis and quality of life of patients.

## Data Availability

Individual participant data are not publicly available because this requirement was not present in the study protocol. The authors had full access to all the data in the study and take responsibility for the integrity of the data and the accuracy of the data analysis. Data may be shared upon request for collaborative studies.

## Supplementary Material

Supplementary material associated with this article can be found, in the online version, at https://doi.org/10.31083/j.rcm2507256.

## References

[1] Heidenreich PA, Bozkurt B, Aguilar D, Allen LA, Byun JJ, Colvin MM (2022). 2022 AHA/ACC/HFSA Guideline for the Management of Heart Failure: Executive Summary: A Report of the American College of Cardiology/American Heart Association Joint Committee on Clinical Practice Guidelines. *Journal of the American College of Cardiology*.

[2] Aboumsallem JP, Moslehi J, de Boer RA (2020). Reverse Cardio-Oncology: Cancer Development in Patients with Cardiovascular Disease. *Journal of the American Heart Association*.

[3] Qiu Y, Jiang P, Huang Y (2023). Anthracycline-induced cardiotoxicity: mechanisms, monitoring, and prevention. *Frontiers in Cardiovascular Medicine*.

[4] Jurczyk M, Król M, Midro A, Kurnik-Łucka M, Poniatowski A, Gil K (2021). Cardiotoxicity of Fluoropyrimidines: Epidemiology, Mechanisms, Diagnosis, and Management. *Journal of Clinical Medicine*.

[5] Dempsey N, Rosenthal A, Dabas N, Kropotova Y, Lippman M, Bishopric NH (2021). Trastuzumab-induced cardiotoxicity: a review of clinical risk factors, pharmacologic prevention, and cardiotoxicity of other HER2-directed therapies. *Breast Cancer Research and Treatment*.

[6] Chaar M, Kamta J, Ait-Oudhia S (2018). Mechanisms, monitoring, and management of tyrosine kinase inhibitors-associated cardiovascular toxicities. *OncoTargets and Therapy*.

[7] Mihalcea D, Memis H, Mihaila S, Vinereanu D (2023). Cardiovascular Toxicity Induced by Vascular Endothelial Growth Factor Inhibitors. *Life (Basel, Switzerland)*.

[8] Varricchi G, Marone G, Mercurio V, Galdiero MR, Bonaduce D, Tocchetti CG (2018). Immune Checkpoint Inhibitors and Cardiac Toxicity: An Emerging Issue. *Current Medicinal Chemistry*.

[9] Conroy RM, Pyörälä K, Fitzgerald AP, Sans S, Menotti A, De Backer G (2003). Estimation of ten-year risk of fatal cardiovascular disease in Europe: the SCORE project. *European Heart Journal*.

[10] Piepoli MF, Hoes AW, Agewall S, Albus C, Brotons C, Catapano AL (2016). 2016 European Guidelines on cardiovascular disease prevention in clinical practice: The Sixth Joint Task Force of the European Society of Cardiology and Other Societies on Cardiovascular Disease Prevention in Clinical Practice (constituted by representatives of 10 societies and by invited experts)Developed with the special contribution of the European Association for Cardiovascular Prevention & Rehabilitation (EACPR). *European Heart Journal*.

[11] Reddy P, Shenoy C, Blaes AH (2017). Cardio-oncology in the older adult. *Journal of Geriatric Oncology*.

[12] Yeh ETH, Bickford CL (2009). Cardiovascular complications of cancer therapy: incidence, pathogenesis, diagnosis, and management. *Journal of the American College of Cardiology*.

[13] Barachini S, Ghelardoni S, Varga ZV, Mehanna RA, Montt-Guevara MM, Ferdinandy P (2023). Antineoplastic drugs inducing cardiac and vascular toxicity - An update. *Vascular Pharmacology*.

[14] Lyon AR, Dent S, Stanway S, Earl H, Brezden-Masley C, Cohen-Solal A (2020). Baseline cardiovascular risk assessment in cancer patients scheduled to receive cardiotoxic cancer therapies: a position statement and new risk assessment tools from the Cardio-Oncology Study Group of the Heart Failure Association of the European Society of Cardiology in collaboration with the International Cardio-Oncology Society. *European Journal of Heart Failure*.

[15] Chow SL, Maisel AS, Anand I, Bozkurt B, de Boer RA, Felker GM (2017). Role of Biomarkers for the Prevention, Assessment, and Management of Heart Failure: A Scientific Statement From the American Heart Association. *Circulation*.

[16] Kong ASY, Lai KS, Hee CW, Loh JY, Lim SHE, Sathiya M (2022). Oxidative Stress Parameters as Biomarkers of Cardiovascular Disease towards the Development and Progression. *Antioxidants (Basel, Switzerland)*.

[17] Zhang N, Wang JX, Wu XY, Cui Y, Zou ZH, Liu Y (2022). Correlation Analysis of Plasma Myeloperoxidase Level with Global Registry of Acute Coronary Events Score and Prognosis in Patients With Acute Non-ST-Segment Elevation Myocardial Infarction. *Frontiers in Medicine*.

[18] Lv X, Pan C, Guo H, Chang J, Gao X, Wu X (2023). Early diagnostic value of high-sensitivity cardiac troponin T for cancer treatment-related cardiac dysfunction: a meta-analysis. *ESC Heart Failure*.

[19] de Boer RA, Nayor M, deFilippi CR, Enserro D, Bhambhani V, Kizer JR (2018). Association of Cardiovascular Biomarkers With Incident Heart Failure With Preserved and Reduced Ejection Fraction. *JAMA Cardiology*.

[20] Lazar AA, Cole BF, Bonetti M, Gelber RD (2010). Evaluation of treatment-effect heterogeneity using biomarkers measured on a continuous scale: subpopulation treatment effect pattern plot. *Journal of Clinical Oncology: Official Journal of the American Society of Clinical Oncology*.

[21] Curigliano G, Lenihan D, Fradley M, Ganatra S, Barac A, Blaes A (2020). Management of cardiac disease in cancer patients throughout oncological treatment: ESMO consensus recommendations. *Annals of Oncology: Official Journal of the European Society for Medical Oncology*.

[22] deFilippi CR, de Lemos JA, Christenson RH, Gottdiener JS, Kop WJ, Zhan M (2010). Association of serial measures of cardiac troponin T using a sensitive assay with incident heart failure and cardiovascular mortality in older adults. *JAMA*.

[23] Ky B, Putt M, Sawaya H, French B, Januzzi JL, Sebag IA (2014). Early increases in multiple biomarkers predict subsequent cardiotoxicity in patients with breast cancer treated with doxorubicin, taxanes, and trastuzumab. *Journal of the American College of Cardiology*.

[24] Otsuka T, Kawada T, Ibuki C, Seino Y (2010). Association between high-sensitivity cardiac troponin T levels and the predicted cardiovascular risk in middle-aged men without overt cardiovascular disease. *American Heart Journal*.

[25] Sawaya H, Sebag IA, Plana JC, Januzzi JL, Ky B, Tan TC (2012). Assessment of echocardiography and biomarkers for the extended prediction of cardiotoxicity in patients treated with anthracyclines, taxanes, and trastuzumab. *Circulation. Cardiovascular Imaging.*.

[26] Michel L, Rassaf T, Totzeck M (2018). Biomarkers for the detection of apparent and subclinical cancer therapy-related cardiotoxicity. *Journal of Thoracic Disease*.

[27] Dong Y, Wu Q, Hu C (2022). Early Predictive Value of NT-proBNP Combined with Echocardiography in Anthracyclines Induced Cardiotoxicity. *Frontiers in Surgery*.

[28] Kakafika AI, Liberopoulos EN, Mikhailidis DP (2007). Fibrinogen: a predictor of vascular disease. *Current Pharmaceutical Design*.

[29] Lowe GD, Rumley A (1999). Use of fibrinogen and fibrin D-dimer in prediction of arterial thrombotic events. *Thrombosis and Haemostasis*.

[30] Wilhelmsen L, Svärdsudd K, Korsan-Bengtsen K, Larsson B, Welin L, Tibblin G (1984). Fibrinogen as a risk factor for stroke and myocardial infarction. *The New England Journal of Medicine*.

[31] Akgul O, Uyarel H (2013). D-dimer: a novel predictive marker for cardiovascular disease. *International Journal of Cardiology*.

[32] Lowe GDO (2005). Fibrin D-dimer and cardiovascular risk. *Seminars in Vascular Medicine*.

[33] Onitilo AA, Engel JM, Stankowski RV, Liang H, Berg RL, Doi SAR (2012). High-sensitivity C-reactive protein (hs-CRP) as a biomarker for trastuzumab-induced cardiotoxicity in HER2-positive early-stage breast cancer: a pilot study. *Breast Cancer Research and Treatment*.

[34] Chien SC, Chen CY, Leu HB, Su CH, Yin WH, Tseng WK (2017). Association of low serum albumin concentration and adverse cardiovascular events in stable coronary heart disease. *International Journal of Cardiology*.

[35] Matsushita K, Coresh J, Sang Y, Chalmers J, Fox C, Guallar E (2015). Estimated glomerular filtration rate and albuminuria for prediction of cardiovascular outcomes: a collaborative meta-analysis of individual participant data. *The Lancet. Diabetes & Endocrinology.*.

[36] Royston P, Altman DG, Sauerbrei W (2006). Dichotomizing continuous predictors in multiple regression: a bad idea. *Statistics in Medicine*.

[37] Cardinale D, Sandri MT, Martinoni A, Tricca A, Civelli M, Lamantia G (2000). Left ventricular dysfunction predicted by early troponin I release after high-dose chemotherapy. *Journal of the American College of Cardiology*.

[38] Pavo N, Raderer M, Hülsmann M, Neuhold S, Adlbrecht C, Strunk G (2015). Cardiovascular biomarkers in patients with cancer and their association with all-cause mortality. *Heart (British Cardiac Society)*.

[39] Blaes AH, Rehman A, Vock DM, Luo X, Menge M, Yee D (2015). Utility of high-sensitivity cardiac troponin T in patients receiving anthracycline chemotherapy. *Vascular Health and Risk Management*.

[40] Menzel A, Samouda H, Dohet F, Loap S, Ellulu MS, Bohn T (2021). Common and Novel Markers for Measuring Inflammation and Oxidative Stress Ex Vivo in Research and Clinical Practice-Which to Use Regarding Disease Outcomes?. *Antioxidants (Basel, Switzerland)*.

[41] Steven S, Frenis K, Oelze M, Kalinovic S, Kuntic M, Bayo Jimenez MT (2019). Vascular Inflammation and Oxidative Stress: Major Triggers for Cardiovascular Disease. *Oxidative Medicine and Cellular Longevity*.

[42] Oguntibeju OO (2019). Type 2 diabetes mellitus, oxidative stress and inflammation: examining the links. *International Journal of Physiology, Pathophysiology and Pharmacology*.

[43] Fernández-Sánchez A, Madrigal-Santillán E, Bautista M, Esquivel-Soto J, Morales-González A, Esquivel-Chirino C (2011). Inflammation, oxidative stress, and obesity. *International Journal of Molecular Sciences*.

[44] Fernández-García JC, Cardona F, Tinahones FJ (2013). Inflammation, oxidative stress and metabolic syndrome: dietary modulation. *Current Vascular Pharmacology*.

[45] Palomo IG, Gutiérrez CL, Alarcón ML, Jaramillo JC, Segovia FM, Leiva EM (2009). Increased concentration of plasminogen activator inhibitor-1 and fibrinogen in individuals with metabolic syndrome. *Molecular Medicine Reports*.

[46] Ryu HH, Ahn SH, Kim SO, Kim JE, Kim JS, Ahn JH (2021). Comparison of metabolic changes after neoadjuvant endocrine and chemotherapy in ER-positive, HER2-negative breast cancer. *Scientific Reports*.

[47] Giskeødegård GF, Madssen TS, Sangermani M, Lundgren S, Wethal T, Andreassen T (2022). Longitudinal Changes in Circulating Metabolites and Lipoproteins After Breast Cancer Treatment. *Frontiers in Oncology*.

[48] Ladas EJ, Jacobson JS, Kennedy DD, Teel K, Fleischauer A, Kelly KM (2004). Antioxidants and cancer therapy: a systematic review. *Journal of Clinical Oncology: Official Journal of the American Society of Clinical Oncology*.

[49] Clasen SC, Scherrer-Crosbie M (2018). Applications of left ventricular strain measurements to patients undergoing chemotherapy. *Current Opinion in Cardiology*.

[50] Sławiński G, Hawryszko M, Liżewska-Springer A, Nabiałek-Trojanowska I, Lewicka E (2023). Global Longitudinal Strain in Cardio-Oncology: A Review. *Cancers*.

[51] Neves KB, Rios FJ, van der Mey L, Alves-Lopes R, Cameron AC, Volpe M (2018). VEGFR (Vascular Endothelial Growth Factor Receptor) Inhibition Induces Cardiovascular Damage via Redox-Sensitive Processes. *Hypertension (Dallas, Tex.: 1979)*.

[52] Cohen JB, Brown NJ, Brown SA, Dent S, van Dorst DCH, Herrmann SM (2023). Cancer Therapy-Related Hypertension: A Scientific Statement from the American Heart Association. *Hypertension (Dallas, Tex.: 1979)*.

[53] Koene RJ, Prizment AE, Blaes A, Konety SH (2016). Shared Risk Factors in Cardiovascular Disease and Cancer. *Circulation*.

[54] Oikawa M, Yaegashi D, Yokokawa T, Misaka T, Sato T, Kaneshiro T (2022). D-Dimer Is a Predictive Factor of Cancer Therapeutics-Related Cardiac Dysfunction in Patients Treated with Cardiotoxic Chemotherapy. *Frontiers in Cardiovascular Medicine*.

[55] Alexandraki A, Papageorgiou E, Zacharia M, Keramida K, Papakonstantinou A, Cipolla CM (2023). New Insights in the Era of Clinical Biomarkers as Potential Predictors of Systemic Therapy-Induced Cardiotoxicity in Women with Breast Cancer: A Systematic Review. *Cancers*.

[56] Ananthan K, Lyon AR (2020). The Role of Biomarkers in Cardio-Oncology. *Journal of Cardiovascular Translational Research*.

