# Supplementary material for: Serum Biomarkers to Dynamically Predict the Risk of Cardiovascular Events in Patients under Oncologic Therapy. A Multicenter Observational Study
Source: Rev Cardiovasc Med. 2024 Jul 9;25(7):256. doi: 10.31083/j.rcm2507256 (PMC11317344; doi:10.31083/j.rcm2507256)
Supplement: Supplementary file 1 [file 2153-8174-25-7-256-s1.docx]

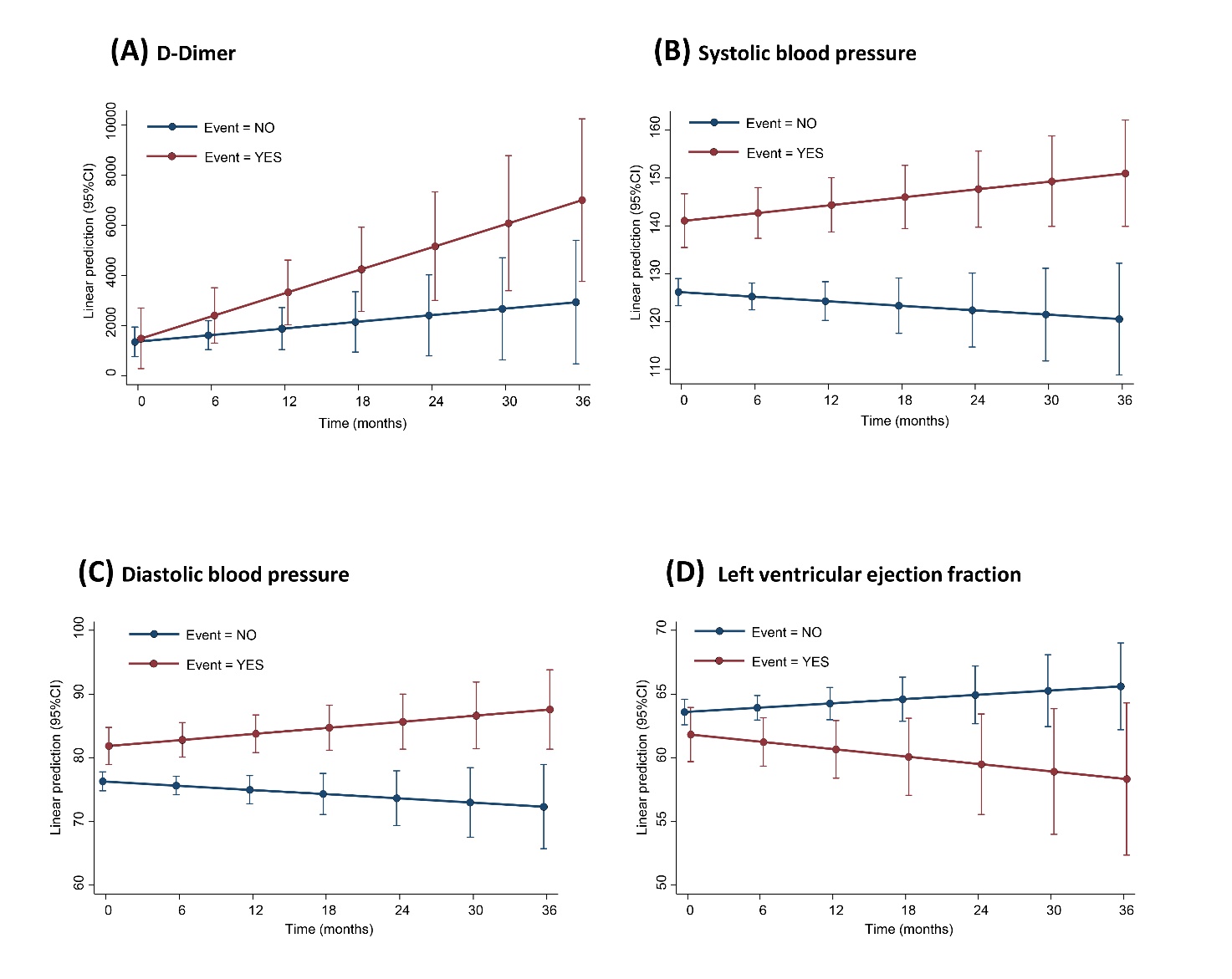
**Supplementary Figure 1:** Marginal linear predicted means from repeated measure mixed models of (A) D-Dimer, (B) Systolic blood pressure, (C) Diastolic blood pressure and (D) LVEF in subjects who did/did not experienced CV event

**Supplementary Table 1: Inclusion and exclusion criteria**

| **Inclusion** | **Exclusion** |
| --- | --- |
| Age > 75 years or age ≤ 75 with high or moderate CV risk according to ESC guidelines | Age ≤ 75 with low CV risk according to ESC guidelines |
| Diagnosis of early or metastatic solid tumor for which is indicated treatment with at least one of these antineoplastic drugs: anthracyclines, docetaxel, paclitaxel, cisplatin, 5FU, capecitabine, trastuzumab, bevacizumab, pertuzumab, sorafenib, sunitinib, pazopanib, lapatinib, axitinib, regorafenib, everolimus, tamoxifen and abiraterone.  Previous anticancer treatment with any other drug which is not listed above was admitted. | Earlier than 10 years treatment with any one of these antineoplastic drugs: anthracyclines, docetaxel, paclitaxel, cisplatin, 5FU, capecitabine, trastuzumab, bevacizumab, pertuzumab, sorafenib, sunitinib, pazopanib, lapatinib, axitinib, regorafenib, everolimus, tamoxifen and abiraterone |
| Normal cardiac function, i.e. asymptomatic patients with LVEF ≥ 50% | Abnormal cardiac function (i.e. LVEF ≤ 50% and/or ongoing CV diseases, such as heart failure, uncontrolled arterial hypertension, unstable angina pectoris, cardiac arrhythmias associated with CV instability and symptoms or psychiatric diseases |
| Presence of signed informed consent | Absence of signed informed consent |

Abbreviations: CV: cardiovascular; ESC: European Society of Cardiology; 5-FU: 5-fluorouracil; LVEF: Left ventricular ejection fraction.

| **Supplementary Table 2. Definition of the cardiovascular event.** |
| --- |
| The cardiovascular event (primary endpoint) was defined as the occurrence of at least one of the following conditions:   - death due to CV disease; - confirmed onset of major ischemic CV disease (e.g.: stroke, TIA, AMI, unstable angina, peripheral acute arterial ischemia); - confirmed onset of heart failure (cardiologic visit and instrumental exams) which causes interruption of antineoplastic treatment; - confirmed onset of hypertension: Systolic Blood Pressure ≥150 mmHg or Diastolic Blood Pressure ≥100mmHg) which causes interruption of antineoplastic treatment*; - hospitalization for CV diseases; - decrease in the LVEF of > 10 percentage points to a value <50% [14]; - confirmed onset of kidney damage (eGFR calculated by using Cockcroft’s method): absolute reduction of eGFR below 30% mL/min/1.73m2; relative reduction of eGFR >30% in comparison with baseline value; serious increase in albuminuria (ACR ≥300 mg/g). |

Abbreviations and notes: CV= cardiovascular; TIA = Transient Ischaemic Attack, AMI= Acute Myocardial Infarction; LVEF = Left Ventricular Ejection Function; eGFR= estimated Glomerular Filtration Rate; ACR=Albumin-to-creatinine ratio; * according to Common Terminology Criteria for Adverse Events (CTCAE) V 3.0. Available online: http://ctep.cancer.gov/protocolDevelopment/electronic_applications/docs/ctcaev3.pdf

|  | **Supplementary Table 3: CV events occurred (n=28)** | |
| --- | --- | --- |
|  | **Event** | **Number of patients (%)** |
|  | Arterial hypertension | 13 (43.4) |
|  | LVEF reduction | 3 (10.7) |
|  | Peripheral Venous Thrombosis | 3 (10.7) |
|  | Pulmonary Embolism | 3 (10.7) |
|  | Heart failure | 3 (10.7) |
|  | Death | 2 (7.1) |
|  | TIA | 1 (3.6) |
|  | Abbreviations: CV= cardiovascular; LVEF = Left Ventricular Ejection Function; TIA = transient ischaemic attack | |

| **Supplementary Table 4: Descriptive statistics of biomarkers levels at baseline.** | | | |
| --- | --- | --- | --- |
|  | mean (SD) | median (IQR) | Skewness, Kurtosis |
| Total Cholesterol, mg/dL | 193 (47) | 189 (163-215) | 1.26, 7.73 |
| LDL Cholesterol, mg/dL | 117 (73) | 108 (83-134) | 6.14, 54.63 |
| HDL Cholesterol, mg/dL | 56 (19) | 53 (43-68) | 0.99, 4.92 |
| Triglycerides, mg/dL | 130 (61) | 119 (92-150) | 2.02, 9.42 |
| Glycemia, mg/dL | 107 (30) | 98 (87-118) | 1.82, 7.20 |
| Microalbuminuria, mg/die | 35 (106) | 5.5 (1.9-16.4) | 4.68, 26.11 |
| Creatinine, mg/dL | 17.5 (39.2) | 0.9 (0.8-1.2) | 2.43, 8.45 |
| Troponin T/I (hs), pg/mL | 11.7 (11.4) | 9.7 (6.0-13.1) | 4.63, 30.80 |
| BNP, pg/mL | 270 (405) | 137 (70-295) | 4.77, 33.4 |
| ACR, mg/mMol | 24.4 (100.3) | 2.4 (0.8-10.0) | 6.99, 53.3 |
| eGFR, ml/min | 73.2 (23.9) | 72.9 (55.5-89.0) | 0.93, 5.22 |
| Fibrinogen, mg/dL | 459 (155) | 419 (345-541) | 1.41, 5.36 |
| D-Dimer, ng/mL | 1308 (1142) | 890 (492-1840) | 1.79, 6.32 |
| C-Reactive Protein, mg/L | 8.4 (24.6) | 0.6 (0.2-2.6) | 4.63, 28.1 |
| Abbreviations: SD= standard deviation; IQR= interquartile range; LDL=low density lipoprotein; HDL=high density lipoprotein; BNP: brain natriuretic peptide; ACR=Albumin-to-creatinine ratio; eGFR: estimated Glomerular Filtration Rate. | | | |

| **Supplementary Table 5:** **Main subjects’ characteristics, by fibrinogen median value (419 mg/dL) at baseline** | | | |
| --- | --- | --- | --- |
|  | ≤ 419 mg/dL | > 419 mg/dL | P-value |
| Age | 71.7 (8.3) | 70.4 (9.2) | 0.478 |
|  |  |  |  |
| Sex |  |  |  |
| Female | 35 (71.4%) | 24 (52.2%) | 0.053 |
| Male | 14 (28.6%) | 22 (47.8%) |  |
|  |  |  |  |
| Tumor Type |  |  |  |
| Colorectal | 14 (28.6%) | 9 (19.6%) | 0.306 |
| Breast | 21 (42.9%) | 8 (17.4%) | 0.007 |
| Lung | 1 (2.0%) | 3 (6.5%) | 0.277 |
| Kidney | 1 (2.0%) | 7 (15.2%) | 0.021 |
| Ovary | 4 (8.2%) | 3 (6.5%) | 0.760 |
| HCC | 0 (0.0%) | 2 (4.3%) | 0.140 |
|  |  |  |  |
| ECOG PS 1 | 1 (2.2%) | 9 (23.1%) | 0.003 |
|  |  |  |  |
| Stage of cancer |  |  |  |
| In situ | 22 (44.9%) | 11 (23.9%) | 0.075 |
| Locally advanced | 11 (22.4%) | 11 (23.9%) |  |
| Metastatic | 16 (32.7%) | 24 (52.2%) |  |
|  |  |  |  |
| Body Mass Index, kg/m^2^ | 25.2 (4.2) | 25.5 (4.4) | 0.789 |
|  |  |  |  |
| Cardiovascular risk |  |  |  |
| Low | 9 (18.4%) | 9 (19.6%) | 0.096 |
| Medium | 25 (51.0%) | 14 (30.4%) |  |
| High | 15 (30.6%) | 23 (50.0%) |  |
|  |  |  |  |
| Previous antineoplastic treatment |  |  |  |
| No | 38 (77.6%) | 40 (87.0%) | 0.232 |
| Yes | 11 (22.4%) | 6 (13.0%) |  |
| Mean (Standard Deviation) or n (%) | | | |
